# Limb salvage in osteosarcoma using autoclaved tumor-bearing bone

**DOI:** 10.1186/1477-7819-10-105

**Published:** 2012-06-08

**Authors:** Kok Long Pan, Wai Hoong Chan, Gek Bee Ong, Shanmugam Premsenthil, Mohammad Zulkarnaen, Dayangku Norlida, Zainal Abidin

**Affiliations:** 1 Department of Orthopaedics, Faculty of Medicine and Health Sciences, Universiti Malaysia Sarawak, Sarawak General Hospital, Kuching, Sarawak, Malaysia; 2 Department of Paediatric Oncology, Sarawak General Hospital, Kuching, Sarawak, Malaysia; 3 Department of Orthopaedics (Oncology), Faculty of Medicine and Health Sciences, Universiti Malaysia Sarawak, Sarawak General Hospital, Kuching, Sarawak, Malaysia; 4 Department of Pathology, Faculty of Medicine and Health Sciences, Universiti Malaysia Sarawak, Sarawak General Hospital, Kuching, Sarawak, Malaysia

## Abstract

****Background**:**

Tumor prostheses currently give the best short- and medium-term results for limb-salvage reconstruction procedures in the treatment of bone tumors. However, in developing countries, the cost of a tumor prosthesis is beyond the reach of much of the population. We report the use of autoclaved tumor-bearing bone in 10 patients, as an affordable alternative to the use of prostheses.

****Methods**:**

This is a case series of 10 patients (mean age 19 years) with osteosarcoma who were treated at our hospital from 1998 to 2008, and followed up for a mean of 35 months (range 14 to 8). The femur was involved in six cases, the humerus in three cases, and the ulna in one case. The mean length of the autoclaved bone was 150 mm (range 60–210).

****Results**:**

Bone union occurred in seven patients over an mean duration of 12 months (range 8–17). Three patients had non-union. Two of these had associated implant failure, with one of them also developing chronic infection, and the third is still being followed up. Two other patients had local recurrence.

****Conclusion**:**

The use of autoclaved tumor grafts provides an inexpensive limb-salvage option without sacrificing appropriate oncologic principles. A painless and stable limb is achievable, and the use of this technique can be further refined.

## **Background**

After many decades of limb-salvage surgery for bone tumors, it seems clear that tumor prostheses give the best results in terms of function and comparative paucity of complications, at least in the short and medium term [1]. Most studies on tumor prostheses have been carried out in centers located in wealthier countries, and in poorer countries, these prostheses are simply not affordable. According to the World Health Organization, 80% of the world’s population live in developing countries, thus the availability and affordability of these state-of-the-art techniques for these populations is limited [2]. For example, in our own hospital, the cost of a prosthesis is equivalent to 2 years’ wages for the average patient. Thus, even though limb salvage is often possible, amputation is carried out because the patient is unable to afford the alternative [1,3],.

In this study, we used autoclaved tumor-bearing bone as an affordable alternative to a prosthesis, requiring only the material and equipment used in fracture treatment. For tumors in the upper limb, the autoclaved bone acts as a spacer, whereas for the lower limb, it is used mainly for fusion at the knee joint. Initial complication rates of this technique are higher, but once union is achieved, the function of the salvaged limb is acceptable and achieves better results than with amputations of the arm with loss of the hand or with above-knee amputations of the leg [4]. It also provides a stable lower limb , and avoids the common sequelae of loosening of a prosthesis [5].

## **Methods**

This was a retrospective case series. From 1998 to 2008, 18 patients with primary malignant tumors involving the limb bones were treated with wide excision and reconstruction using the patients own bone tissue, which was removed during tumor excision, autoclaved, and reimplanted. The university research and ethics committee approved the grant and methodology for the study. Informed consent was obtained from the patients or parents for publication and use of images. Of the eighteen patients, four patients died within 1 year of surgery (two as a result of rapidly progressing lung metastases and another two from causes related to the complications of chemotherapy), and another four patients were lost to follow-up. These eight patients were excluded, leaving 10 patients (four male and six female patients; mean age 19 years, range 11–35), who were followed up for a mean of 35 months (range 14–80) (Table 1).

**Table 1:** Clinical and tumor characteristics, treatment, and follow-up outcomes

**Patient**	**Age/ gender**	**Diagnosis/ site**	**Tumor size, mm**^**1**^	**Surgery**	**Length of autoclaved bone. mm**	**Follow-up, months**	**Results/time to union, months**	**Complications**
1	14/F	OS/distal femur	30/50/100	Knee fusion, IMN	180	80	Non-union, 31	Infection
2	14/M	OS/distal femur	60/60/150	Knee fusion, locking plate	210	14	14	
3	15/F	OS/proximal humerus	70/80/200	Spacer, cement graft rod, composite	80	15	8	Fracture, local recurrence
4	11/F	OS/proximal humerus	50/110/150	Spacer, cement graft rod, composite	80	14	10	
5	17/F	OS, distal femur	150/200	Knee fusion, IMN, plate	200	66	Non-union, 42	
6	13/M	OS/proximal humerus	110	Spacer, cement graft rod, composite	170	42	11	
7	32/F	OS/distal femur	100/60/70	Knee fusion, locking plate	180	25	9	Infection
8	35/F	Soft-tissue OS, ulna	60/50/20	Plate	60	55	15	
9	20/M	OS/distal femur	110/90/70	Knee fusion, plate	190	19	17	Local recurrence
10	23/M	OS/distal femur	110/100/140	Knee fusion, locking plate	170	17	Non-union, 17^2^	

Abbreviations: *IMN*, intramedullary nail; *OS*, osteosarcoma.

^1^Tumor size: anterior–posterior/width/length.

^2^Patient is still being followed up.

**Figure 1:**
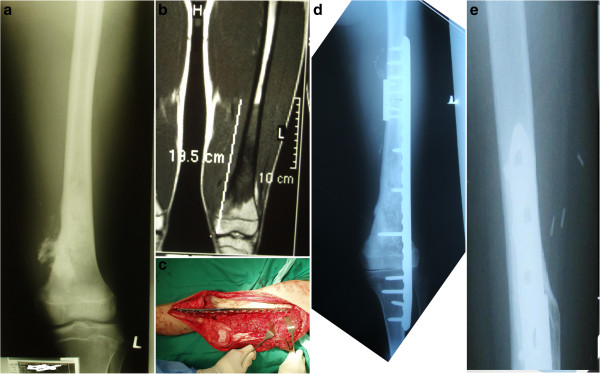
**Patient 2: 14-year-old boy with osteosarcoma of the distal femur. (A)** Plain radiograph showing changes at the distal femur. **(B)** Magnetic resonance imaging scan showing the intramedullary extent of the tumor (180 mm). A further 30 mm was resected proximally during surgery, making a total of 210 mm of bone that was autoclaved and replaced. **(C)** Intra-operative photograph showing the autoclaved tumor bone placed in its original bed. **(D)** Post-operative radiograph showing fixation of the autoclaved tumor bone with a long locking plate and a 3.5 dynamic compression plate at the proximal junction. **(E)** Follow-up 14 months later; bone union and full weight-bearing without aids.

Nine patients had a primary malignant bone tumor (all osteosarcomas) and one patient had a soft-tissue osteosarcoma that had invaded the adjacent bone. Six tumors involved the distal femur (Figure 1), three involved the proximal humerus, and one involved the middle third of the ulna. All 10 patients underwent staging with plain radiographs, magnetic resonance imaging (MRI) scan (of the whole bone to identify any skip metastasis), computed tomography of the thorax, and a bone scan. Examination of an incisional biopsy was used to confirm the diagnosis. All cancers were high-grade and extra-compartmental tumors. Treatment was started with three cycles of neoadjuvant chemotherapy, with an interval of 3 weeks between each cycle. For each cycle, patients were given cisplatin on day 1, and adriamycin on days 1–3. Each cycle of chemotherapy was followed by another MRI assessment, and surgery was scheduled when limb salvage was deemed feasible.

During the surgery, wide excision was performed, and the soft-tissue components were grossly removed. The specimen was then heated in an autoclave at 120° C for 10 minutes. Upon removal from the autoclave, the remaining soft tissue and joint cartilage were easily scraped off from the surface of the bone, Any ‘new’ bone formed by the tumor osteoblastic process had been softened by the autoclaving procedure and this was also scraped off easily. The specimen was then cleaned with normal saline and prepared for reinsertion. The whole process was performed under sterile conditions, with sterile wraps used for transport between the surgical field and the autoclave.

The autoclaved bone was reinserted in the original tumor bed, and held in place by plates or intramedullary nails. For procedures involving the upper limb, bone cement was used as an added spacer in regions where the tumor had destroyed the bone. The mean length of autoclaved bone was 150 mm (range 6–21), and the margin of normal bone removed beyond the tumor was 30mm. The resection margin for the soft tissue was through normal-appearing tissue, generally 10–20 mm beyond the tumor, except in regions where vital neurovascular bundles abutted the tumor. An intra-operative biopsy was taken of the intramedullary marrow proximal or distal to the tumor. Occasionally, biopsies were also taken from the tumor bed in areas where the resection margin was deemed by the surgeon to be suspect. Tumor tissue removed before autoclaving was sent for further histopathological examination, including evaluation of the percentage of necrosis.

Once the wounds had healed, patients were given another three cycles of adjuvant chemotherapy. Patients with tumors in the lower limb were allowed to ambulate with crutches, starting from non-weight-bearing and graduated partial weight-bearing, and progressing to full weight-bearing over the subsequent months, after union was ascertained from radiographs. Patients with tumors in the arm used a triangular bandage for 6 weeks for limb support, after which they were encouraged to freely use the limb as pain allowed.

Patients were initially followed up at intervals of 6 weeks then every 3 months until 1.5 years after surgery, then, every 6 months until 5 years after surgery, and then yearly thereafter.

At each follow-up, plain radiographs (two views) were taken, encompassing both the proximal and distal graft-host junction (when this applied). All readings of the radiographs in relation to union were done by the same investigator (KLP). Union was judged to have taken place when bridging callus was seen on three cortices. However, this was not always easily visualized when the junction was bridged by two plates or by a nail plus a plate. In such instances, two cortices with bridging calluses were accepted for union. For reconstructions with two graft-host junctions, the time to union was the time taken for both junctions to be united. In cases of lower limb surgery with knee fusions, the distal junction (cancellous bone) always united first. Bone grafting was not performed for patients with non-union.

## **Results**

Union occurred in seven of the ten patients over a mean duration of 12 months (range 8–17). Two patients had non-union associated with implant failure; both underwent an additional procedure (one received a plate and one Ilizarov bone transport) which subsequently resulted in union. Union had not been achieved in the final patient at 17 months, and this patient is still being followed up. Bone grafting was contemplated for this patient, but this was not carried out because it was likely that the femoral artery and vein were embedded in the mass of fibrous scar tissue around the non-union site. Some skin had also been removed with the tumor from the first surgery, and tight skin coverage over the bone grafts may predispose to skin necrosis.

In relation to complications, one patient (non-union of the humerus) had a fracture through the autoclaved bone in an area that was not reinforced by any metal, and this also united with an added plate. One patient had an infection, which cleared up with antibiotics, debridement, and a local medial gastrocnemius muscle rotation flap. Another patient (with non-union), developed chronic osteomyelitis and still had a discharging sinus at the most recent follow-up. Two patients had local recurrence of the cancer: one underwent radiotherapy and the other had an amputation.

All biopsies taken from suspected tumor-bed sites and from the bone marrow proximal or distal to the resected parts were clear of tumor cells.

## **Discussion**

Most primary malignant tumors involving the limb bones are surgically treated, either by amputations or limb salvage. Limb salvage requires reconstituting the skeletal defect after tumor resection. The main modes of reconstruction in the lower limb have used resection arthrodesis, osteoarticular allografts, or tumor prostheses [4].

It is generally accepted that tumor prostheses provide the best results in relation to function and complications, at least in the short and medium term [6,7]. However, they are costly and require monitoring in the long term for loosening, which may entail replacement with another costly implant. At our hospital, each of these prostheses is equivalent to 2 years of the average patient’s wages. Allografts are affordable but require a nearby active bone bank and the availability of appropriate bone parts and sizes. Use of allografts also necessitates timely transport and storage at the recipient center before surgery.

In less developed countries, many orthopedic surgeons who seek to perform salvage on the lower limbs in these patients have to fall back on resection arthrodesis, which has been shown to be functionally superior to an above-knee amputation [4]. When an amputation is the only option offered, many patients and parents will not consent to ablative surgery and will voluntarily discharge themselves from hospital to seek traditional treatment. There is thus a need to develop and refine methods such as resection arthrodesis, which is affordable and, at the same time, does not compromise the principles of treatment of such patients.

In patients with high-grade osteosarcoma of the proximal humerus, limb resection in lieu of a forequarter amputation is widely accepted, especially when the elbow and hand function can be preserved [8]. Prostheses or intercalary implants are an option. Allografts or autografts (for example, fibula specimens) are often used to bridge the gap, but large autografts will increase donor site morbidity [9].

In the present study, by autoclaving and reusing the patient’s own bone, we obviated the need to procure an allograft. The fact that the bone fits the original defect is an added advantage of the method. There is also no risk of infection such as HIV or hepatitis from a donor bone. Extracorporeal irradiation (instead of autoclaving) and reimplantation has also been used in previous studies, but this increases the intra-operative logistics and necessitates the ready availability of the radiation oncologist and radiotherapy machine at a specified time. Pasteurization is an alternative that does not weaken the bone to the same extent as with autoclaving [10]; it requires the availability of a homeothermal heater to maintain a temperature of 60°C for 30 minutes. Occasionally, it may be found that the tumor has caused large defects or destroyed the bone altogether; in such cases, the defects can be filled or the part refashioned using bone cement.

In our study, union was achieved in seven of the ten patients after a mean duration of 12 months. Similar studies have reported union in 11 out of 12 patients after 24 months [11] and in 11 out of 12 after 4.6 months [12]. Chang *et al*. [13] reported union in 11 out of 14 patients (duration not reported, but in that study, a vascularized bone graft was added to the autoclaved bone.

Two of the non-unions occurred in the femur at the proximal graft-host junction. Both were spanned by intramedullary nails, which did not provide sufficient stability for union, resulting in implant failure as well. We treated one patient by the addition of a plate, and in the other patient, an Ilizarov transport fixator was used because the patient also had an infection. Both bones subsequently united. Having learnt from these cases, we now use a long locking plate spanning both junctions, and add a second four-hole compression plate at the proximal junction, which provides a stronger and more stable construct. We believe that the other important factor in non-union is the vascularity of the overlying soft tissue, particularly the muscle cover, especially at the graft-host junction.

We did not have problems with union in any of the patients who had tumors of the upper limb. However, one patient did sustain a fracture through the autoclaved tumor bone that was not spanned by an implant. After fixation with an additional plate, the fracture united, even though one end of the fracture was part of the autoclaved bone.

Two patients had deep infection. In one patient, the infection cleared after antibiotics, debridement, and covering of the area with a gastrocnemius muscle local rotation flap. We are now careful at the initial surgery to cover all parts of the autoclaved bone with muscle before suturing the skin over it. In the distal femur, this is usually achieved by releasing the sartorius muscle and when necessary, by rotating the gastrocnemius muscle. We treated the other patient with an infected non-union by means of an Ilizarov fixator and bone transport. Although we achieved union, we were not able to clear the infection in this patient.

There were two patients with local recurrence. The recurrent nodules were clinically mobile and arose from the soft tissue rather than from the autoclaved bone. One patient was treated with radiotherapy and the other underwent amputation.

## **Conclusion**

The management of primary malignant bone tumors in less developed countries is often a daunting task, strewn with a long list of complications. Patients often present late, by which time the tumors have grown to a large size, and further delays are encountered in waiting for investigations [14]. The final problem lies in reconstruction of the bone defect with affordable and available means; for many patients at this point, amputation often seems to be the only resort.

The use of autoclaved tumor grafts provides a limb-salvage option that is inexpensive and independent of external resources without sacrificing appropriate oncologic principles. A painless and stable limb is achievable when the short- and medium-term complications can be averted or surmounted.

## **Competing interests**

All authors declare that they have no competing interests.

## Authors’ contribution

KLP was the main surgeon. WHC was the second surgeon. GBO gave chemotherapy for pediatric patients. SP gave chemotherapy for adult patients and also radiotherapy. MZ, DN and ZA red the histological slides before and after the surgical resections. All authors’ read and approved the final manuscript.
